# Transcriptomic profiling of mature embryo from an elite super-hybrid rice LYP9 and its parental lines

**DOI:** 10.1186/1471-2229-8-114

**Published:** 2008-11-11

**Authors:** Xiaomeng Ge, Weihua Chen, Shuhui Song, Weiwei Wang, Songnian Hu, Jun Yu

**Affiliations:** 1 CAS Key Laboratory of Genome Sciences and Information, Beijing Institute of Genomics, Chinese Academy of Sciences, Beijing, 100029, PR China; 2 Graduate University of Chinese Academy of Sciences, Beijing, 100049, PR China

## Abstract

**Background:**

The mature embryo of rice (*Oryza sativa, L*.) is a synchronized and integrated tissue mass laying the foundation at molecular level for its growth, development, and differentiation toward a developing and ultimately a mature plant. We carried out an EST (expressed-sequence-tags)-based transcriptomic study, aiming at gaining molecular insights into embryonic development of a rice hybrid triad–an elite hybrid rice *LYP*9 and its parental lines (*93-11 *and *PA64s*)–and possible relatedness to heterosis.

**Results:**

We generated 27,566 high-quality ESTs from cDNA libraries made from mature rice embryos. We classified these ESTs into 7,557 unigenes (2,511 contigs and 5,046 singletons) and 7,250 (95.9%) of them were annotated. We noticed that the high-abundance genes in mature rice embryos belong to two major functional categories, stress-tolerance and preparation-for-development, and we also identified 191 differentially-expressed genes (General Chi-squared test, *P*-value <= 0.05) between *LYP9 *and its parental lines, representing typical expression patterns including over-dominance, high- and low-parent dominance, additivity, and under-dominance. In *LYP9*, the majority of embryo-associated genes were found not only abundantly and specifically enriched but also significantly up-regulated.

**Conclusion:**

Our results suggested that massively strengthening tissue-(or stage-) characteristic functions may contribute to heterosis rather than a few simple mechanistic explanations at the individual gene level. In addition, the large collection of rice embryonic ESTs provides significant amount of data for future comparative analyses on plant development, especially for the important crops of the grass family.

## Background

Heterosis is a phenomenon that particular inbred lines can produce progenies with favourable phenotypes over their parents, such as stronger tolerance to stresses and higher yields. Two fundamental hypotheses for heterosis were defined in classical genetic studies, including genome-wide dominance complementation and locus-specific over-dominant effects [1-3]. Evidence showed that both play roles in heterosis with involvement of epistasis [4-6]. Another theory suggested that heterosis might result from the loss of control of metabolism among heterozygotes [7]. Along with the development of genome-wide expression technology such as EST, SAGE, and microarrays, current technological advancements allow scientists to gain comprehensive information and knowledge on gene expression profiles of particular tissues from hybrids and their parental lines [8]. As it is obvious that differentially-expressed genes (DEGs) among hybrid triads may offer straightforward molecular clues to characterize phenotypic differences and genes responsible for favourable phenotypes, high-throughput gene expression profiling methods have been used for identifying DEGs in recent years. Various DEGs and their expression patterns in hybrids and their parental lines have been defined and analyzed, including over-dominance, high-parent dominance, additivity, low-parent dominance, and under-dominance [9,10]. The distribution of DEGs varies among different samples, which not only reflects the complexity of rice transcriptomes but also points to the possibility where intricate molecular mechanisms may be involved in heterosis.

As both an important cereal crop and a model plant, the rice genome has been sequenced multiple times for its two major subspecies [11,12]. We have been studying rice genome with a particular interest on the molecular mechanism of heterosis as one of the specific aims proposed for the Super-hybrid Rice Genome Project (SRGP). The project focuses on an elite super-hybrid (*Liang-You-Pei-Jiu, LYP9; *which can yield about 20–30% more grains; [13]) and its parental lines (*93-11 *and *PA64s*). Several datasets of DEGs as potential heterosis-associated genes as well as possible molecular mechanisms have been collected from major tissues, such as leaves, roots, and panicles [10,14]. The mature embryo is an important developmental stage in the rice life cycle, as it is a synchronized, undeveloped miniature plant that consists of precursor tissues for root, leaf, and stem. It was reported that its long-lived mRNA reserved in dry plant seeds might, in general, contain a valuable molecular record of embryogenic development and primary biological process [15]. A recent study suggested that the embryo of heterotic F1 possesses certain advantages at a very early developmental stage [16]. Therefore, it is important to know whether the mature embryo deposits heterotic potential for further development, as a genome-wide transcriptomic study for the mature rice embryo of a hybrid triad has not yet been reported to this date.

In this study, we constructed cDNA libraries for mature embryos from *93-11*, *PA64s *and *LYP9*, and randomly sequenced approximately 10,000 ESTs from each library, resulting in 27,566 high-quality ESTs and 7,557 unigenes. We analyzed these ESTs and identified 191 DEGs between *LYP9 *and its parental lines (General Chi-squared test, *P*-value <= 0.05). Assigning the DEGs into functional categories and expression patterns, we realized that multiple modes are at work for heterosis. All our EST sequences were submitted to the NCBI dbEST database under accession number FG943106 to FG970671.

## Results

### EST acquisition

**Figure 1:**
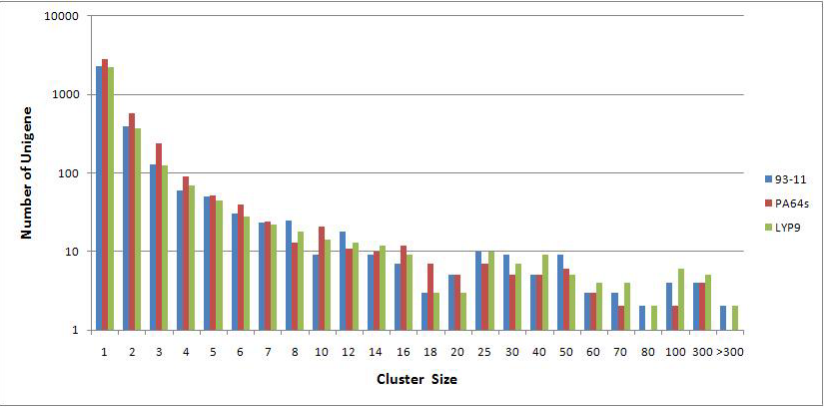
**Unigenes from three libraries sorted by cluster-size**. ESTs from all libraries were clustered into unigenes (contigs and singletons) and the number of unigenes in each size-based cluster bins was plotted.

**Figure 2:**
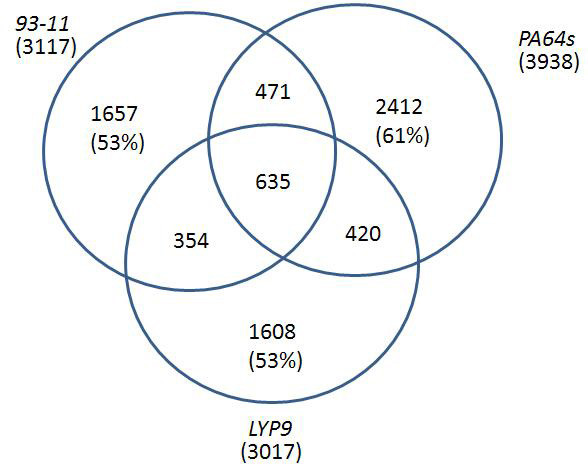
Venn diagrams of unigenes shared among libraries based on unigene counts.

We acquired approximately 10,000 clones from each library, which yielded 28,555 high-quality ESTs after removal of vector and low-quality sequences. The collection was further refined by removing sequences that are not anchored in the *Oryza sativa *Gene Index database (r17) and predicted gene sequences of the *93-11 *genome [11], resulted in 27,566 high-quality ESTs that include 9,084, 9,027, and 9,455 ESTs for *93-11, PA64s, and LYP9*, respectively (Table 1). We assembled the ESTs into 7,557 clusters, including 2,511 contigs and 5,046 singletons. The three datasets contain 3117, 3938, and 3017 unigenes from *93-11*, *PA64s*, and *LYP9*, respectively, with a similar cluster-size distribution (Figure 1). Only 635 (8.4% of total) unigenes were found universally-expressed as they are shared by all three libraries; there are about a third of them are shared by two libraries and more than 50% unigenes in each library are unique (Figure 2). Since most of the unique genes were detected as single copies, we believe that the transcriptome of a mature rice embryo is rather complex and the current sampling is not deep enough to discover low-abundance genes as most of the genes should be shared among the cDNA libraries rather than truly unique to each. Therefore, we focused our analysis on unigenes expressed in relatively high abundance.

**Table 1:** Summary of EST sequences, contigs, and singletons in three rice cDNA libraries

Library ID	No. of reads	No. of high-quality EST	No. of contigs	No. of singletons	No. of unigenes
*93-11*	10875	9084	810	2307	3117
*PA64s*	10751	9027	1127	2811	3938
*LYP9*	11172	9455	784	2233	3017
Total	32798	27566	2511	5046	7557

### Functional annotation

We annotated 7,250 (95.9%) out of a total of 7,557 unigenes in our dataset based on sequence similarity to those in public databases (Table 2). Among 307 un-annotated unigenes, 13 have significant similarity to the repeat sequence collection (TIGR_Oryza_Repeats.v3.3) and one to a non-coding RNA sequence from the mouse genome . The remaining 293 unannotated unigenes are rather short but have open reading frames more than 30 amino acids in length, and they may represent novel protein-coding genes or non-coding RNAs as they are expressed in rice embryos.

**Table 2:** Annotations of unigenes based on different public databases

Database	No. of matched unigenes	Percentage of total unigenes (%)
TIGR OGI(ver17)	7126	94.3
TIGR PseudoMolecule(ver5)	6151	81.4
NCBI UNIGENE(ver62)	6714	88.8
NCBI nr protein database	5831	77.2
*93-11 *BGI_Scan	5854	77.5
Uniprot protein database	3628	48.0
TIGR to GO	4565	60.4
KEGG Automatic Annotation Server	945	12.5

Alignments were performed by using blast (e-value = 1.00e-05), and 7,250 (95.9%) unigenes were annotated.

**Figure 3:**
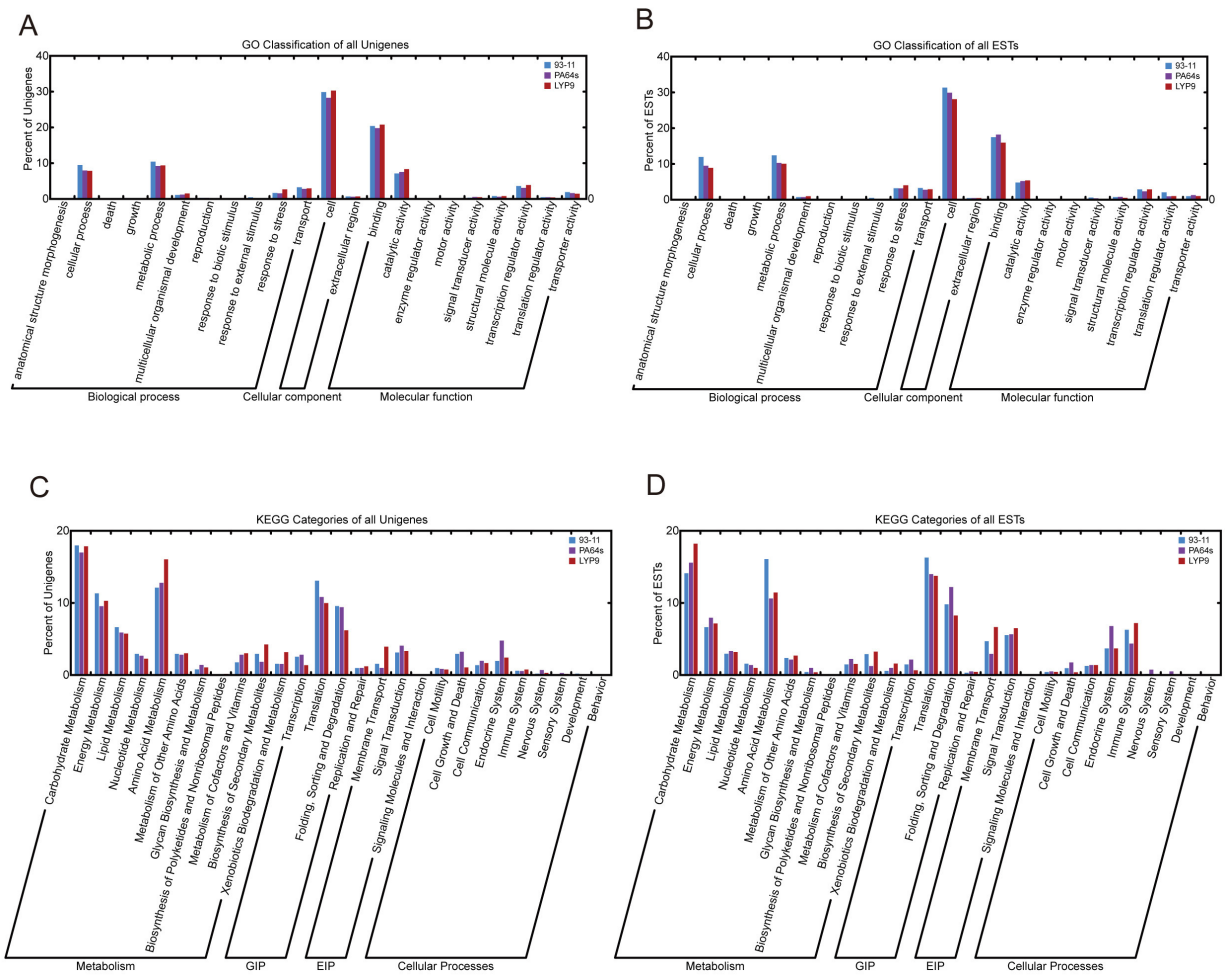
**Functional classification of unigenes**. Functional classifications are based on GO terms (A, unigenes; B, ESTs) and KEGG categories (C, unigenes; D, ESTs).

We aligned the best hits to TIGR OGI database with the Gene Index to GO mapping database at TIGR and assigned at least one GO term to 4,565 (60.4%) transcripts. The GO-annotated unigenes are 1,995 (64.0%), 2,500 (63.5%), and 1,908 (63.2%) for *93-11*, *PA64s*, and *LYP9*, respectively. We assigned most of the unigenes into several functional categories, including cellular process, metabolism process, multicellular organism development, stress tolerance, transport, cell, binding, catalytic activity, transcription regulator activity, translation regulator activity, and transporter activity (Figure 3).

We also annotated our datasets using KEGG Automatic Annotation Server  for pathway information (BBH method). We were able to offer 945 (12.5%) unigenes KO numbers, including 429 (13.8%), 560 (14.2%), and 509 (16.9%) in the libraries of *93-11*, *PA64s*, and *LYP9*, respectively. The categories of metabolism, genetic information processing (GIP), environmental information processing (EIP), and cellular process involved 64.1%, 20.7%, 6.1%, and 9.1% of the total annotated unigenes, respectively. As summarized in Figure 3, carbon (25.9% of metabolism), energy (15.2% of metabolism), and amino acid (22.3% of metabolism) metabolisms are major contributors among the subsets of metabolism. In the category of GIP, translation (43.4% of GIP) and sorting (39.0% of GIP) are the majority as opposed to transcription (11.1% of GIP). Signal transduction (58.1% of EIP) and membrane transport (41.9% of EIP) constitute the majority of EIP. Other than these major categories, the rest of cellular processes showed lower abundance although defence systems (endocrine and immune systems) were found more abundant among the low-abundance categories.

### Expression abundance

We calculated the expression abundance for each unigene using their cluster sizes (a sum of ESTs from all three libraries) and sorted them into three abundance levels: high-abundance (>= 100 ESTs), medium-abundance (>= 13 EST; this cutoff value was chosen as it represents 0.5‰ of the total 27,566 ESTs and is at a turning point when the number of ESTs per unigene was plotted in our redundancy analysis), and low-abundance (Table 3). The top 32 bins of high-abundance genes constitute only 0.42% of total unigenes in kind but 33.47% of the total EST counts (Table 4). The entire collection and its annotated details are listed in Additional file 1: Summary of unigenes. Their sequences are supplemented in Additional file 2: A full list of unigene sequences.

**Table 3:** Classification of unigenes by expression abundance

Category	Standard	Number of genes	Number of ESTs
High	>= 100	32(0.42%)	9227 (33.47%)
Medium	>= 13	183(2.42%)	5315 (19.28%)
Low	< 13	7342(97.16%)	13024 (47.25%)

Alignments were performed by using blast (e-value = 1.00e-05), and 7,250 (95.9%) unigenes were annotated.

**Table 4:** High-abundance genes and functional annotations

Unigene ID	Abundance	TPM^a^	Functional annotation
Unigene_0001	2739	99354	Cytochrome P450 monooxygenase^b^
Unigene_0002	1121	40663	rRNA intron-encoded homing endonuclease
Unigene_0003	443	16069	Os11g0211800
Unigene_0004	379	13748	Em-protein^c^
Unigene_0005	272	9867	Ramy1
Unigene_0006	256	9286	Lectin^b^
Unigene_0007	249	9032	LEA3^b^
Unigene_0008	238	8633	RAB21^b^
Unigene_0009	219	7944	Thaumatin-like protein^b^
Unigene_0010	210	7618	HSP16.9^b^
Unigene_0011	209	7581	HSP70^b^
Unigene_0012	203	7364	HSP17.4^b^
Unigene_0013	197	7146	NAD-/NADP-dependent oxidoreductase
Unigene_0014	171	6203	Late embryogenesis abundant protein D-34^b^
Unigene_0015	164	5949	Senescence-associated protein^b^
Unigene_0016	163	5913	Glyceraldehyde-3-phosphate dehydrogenase
Unigene_0017	161	5840	RAB24^b^
Unigene_0018	151	5477	E2
Unigene_0019	149	5405	EF-1 alpha
Unigene_0020	144	5223	Os03g0723400
Unigene_0021	139	5042	Rab16b^b^
Unigene_0022	138	5006	Ec protein^c^
Unigene_0023	136	4933	Seed maturation protein^b^
Unigene_0024	117	4244	Hypothetical protein APECO1_2466
Unigene_0025	111	4026	Os04g0589800
Unigene_0026	111	4026	Bowman Birk trypsin inhibitor^b^
Unigene_0027	110	3990	RAB25^b^
Unigene_0028	110	3990	HSP90(81-2)^b^
Unigene_0029	109	3954	Multiple stress-responsive zinc-finger protein^b^
Unigene_0030	105	3809	Embryogenic-cell protein 40^c^
Unigene_0031	103	3736	DnaJ protein^b^
Unigene_0032	100	3627	Plasma membrane associated protein

^a^Transcript per million,^b ^stress-tolerance associated genes, and ^c ^development-associated genes.

**Figure 4:**
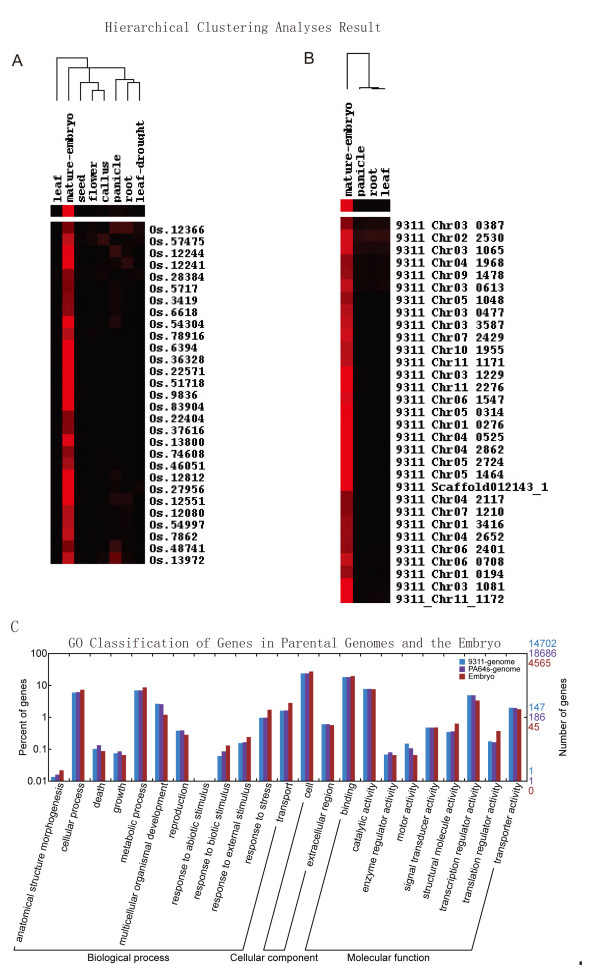
**Hierarchical clustering and GO analyses of embryo-associated genes**. We summarized our hierarchical clustering analyses (A, compared to NCBI DDD database; B, compared to SAGE data generated from the same hybrid line) of high-abundance genes based on data from the embryo and other tissues (also see Table 5). The expression abundance was normalized to transcript per million. The frequencies of unigenes from different libraries are indicated by color intensity (red), and blank was adjusted to be black. A GO classification of embryo-associated genes in comparison to the genome total (C) was also showed to emphasize the fact that the enrichment of stress- and development-associated genes in the mature embryo.

To identify embryo-characteristic genes, we carried out a hierarchical clustering analysis based on EST abundance, rice gene annotation in the unigene database at NCBI, and comparative analysis with representative libraries of other rice tissues selected from the Digital Differential Display (DDD) database (Table 5). We also did a parallel analysis utilizing SAGE data of different tissues (leaf, root, and panicle), which were generated from the same hybrid triad lines [10] based on annotations in our database that contains predicted gene sequences from the *93-11 *genome. The results are rather consistent; not only is there a significant amount of high-abundance genes unique to the mature embryo (Figure 4A and 4B) but also most of the medium-abundance genes are rather characteristic for the embryo (Additional file 3: Hierarchical clustering of medium-abundance genes.). In addition, we compared embryo-associated genes against all predicted genes from the parental genomes (*93-11 *and *PA64s*) based on GO functional classification, and observed the enrichment of genes in several categories, such as response to stimulus and stress in the category Biological Processes (Figure 4C).

**Table 5:** Libraries for Digital-Differential-Display analysis

Tissue	Lib ID	EST	Clustered EST	Unigene
Callus	19058	53637	50361	10713
Flower	19057	46187	43264	10451
9 leaf-stage leaf	19096	13230	12891	681
Drought stress (leaf)	9652	5615	4154	1344
Panicle	19050	13571	13060	3634
Root of seedlings	19053	17250	16565	4445
Seed	19082	9792	9408	4228
***Mature rice embryo***	***local data***	***28555***	***27566***	***7557***

Data from this study are highlighted in bold and italic.

**Figure 5:**
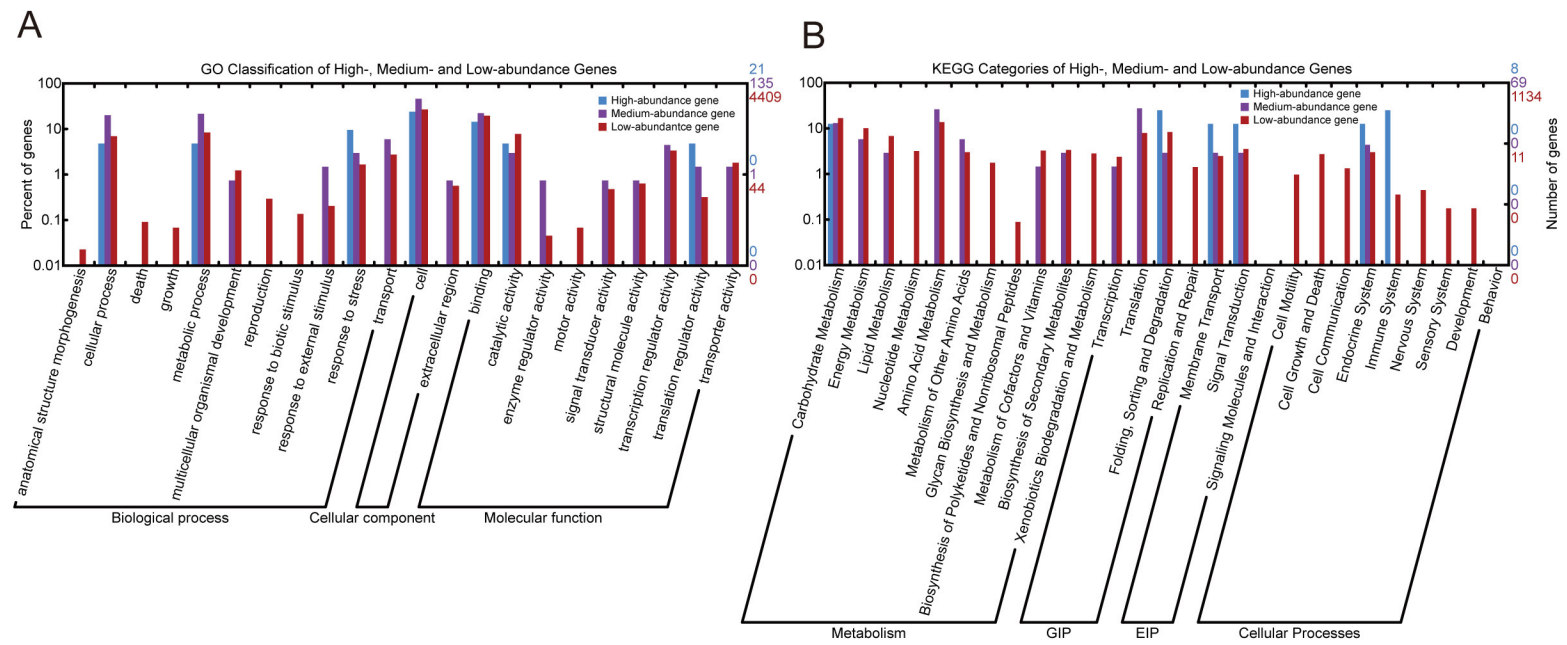
**Functional classification by expression abundance**. GO classification and KEGG pathway analyses based on gene expression abundance. GO classes (A) and KEGG pathways (B) of all unigenes sorted by expression abundance described in Table 3 were shown. The high-abundance genes represent the most important functional categories and pathways in the mature embryo.

Based on GO functional classification, we found that (1) high-abundance genes are enriched in cellular and metabolic processes, response to stress, cell, binding, catalytic activity, and translation regulator activity; (2) medium-abundance genes are those for embryo development, response to external stimulus, transport, enzyme regulator activity, signal transducer activity, structural molecule activity, transcription regulator activity, and transporter activity; and (3) low-abundance genes are involved in anatomical structure morphogenesis, death, growth, reproduction, response to biotic stimulus, and motor activity (Figure 5).

We also analyzed the genes classified as different pathways. We found that (1) high-abundance genes concentrate on carbohydrate metabolism, folding/sorting/degradation, membrane transport, signal transduction, and defence (endocrine and immune) systems, and (2) medium-abundance genes are mainly involved in the pathways of energy metabolism, lipid metabolism, amino acid metabolism, transcription and translation (Figure 5). High-abundance genes were found in the pathways of glycolysis, pores ion channels, protein folding and associated processing, ubiquitin-mediated proteolysis, MAPK signalling, progesterone-mediated oocyte maturation, and antigen processing/presentation; all appear performing important functions in keeping embryonic status and rapid growth recovery.

### Abundant embryo-characteristic gene families

We discovered four major gene families expressed in high-abundance in the rice embryo (Table 6). The most abundant gene family belongs to cytochrome P450 monooxygenases, making up 10% of total ESTs. They are universally-expressed and senescence-associated in all eukaryotic species. Among plants, these enzymes are important for the biosynthesis of hormones, defensive compounds, and fatty acids. The second group is composed of HSPs (heat shock proteins), and they are multifunctional molecular chaperones and many show over-expression during embryonic development and are inducible by desiccation and other stress types. We identified four low-molecular-weight (LMW, 17–30 KD) HSPs in our dataset; they are HSP16.9, HSP17.4, HSP22, and HSP26 as well as four classical HSPs: HSP40, HSP70, HSP90, and HSP101. In numbers, there are 16% of the abundantly-expressed genes and 3.3% of the total ESTs belonging to the HSP family. The third protein family is the LEA (late embryogenesis abundant; 3.0% of the total ESTs) proteins that are a group of proteins accumulating in high abundance during last stages of seed formation and periods of the water shortage in vegetative organs. Ample evidence bsuggested that LEA proteins, especially its subgroup 3 members, are involved in desiccation resistance through a variety of machineries, including water retention, ion sequestration, and direct protein protection [17,18]. The fourth group is the RAB family, which are membrane-associated small GTP-binding proteins localized to discrete subcellular compartments and involved in signal transduction, cytoskeleton organization, and vesicle trafficking. They are also stress-inducible. We identified its four members whose ESTs make up 2.6% of the total collection: RAB16 (b, c, d), RAB21, RAB24, and RAB25.

**Table 6:** Abundant gene families detected in the dataset

Gene family	Subfamily		Expression abundance				Unigene ID
				
			*93-11*	*PA64s*	*LYP9*	Total	
P450^a^	Cytochrome P450 Monooxygenase		1111	348	1280	2739	Unigene_0001

HSP^b^	LMW	Hsp16.9	29	102	79	210	Unigene_0010
		Hsp17.4	28	74	101	203	Unigene_0012
		Hsp22	3	3	13	19	Unigene_0141
		Hsp26	4	6	40	50	Unigene_0055
	Classical HSP protein	Hsp40	29	45	29	103	Unigene_0031
		Hsp70	71	50	88	209	Unigene_0011
		Hsp90	44	34	32	110	Unigene_0027
		Hsp101	1	4	11	16	Unigene_0170

LEA^c^	LEA	LEA3	90	61	98	249	Unigene_0007
		LEA D-34	80	16	75	171	Unigene_0014
		LEA	21	7	27	55	Unigene_0052
		LEA	8	17	13	38	Unigene_0072
		LEA	12	3	19	34	Unigene_0081
	
	Seed maturation protein	PM	61	19	56	136	Unigene_0023
		PM24	8	6	9	23	Unigene_0123
		PM	4	2	11	17	Unigene_0151
		PM	4	4	7	15	Unigene_0180
		PM	3	5	4	12	Unigene_0228
		PM	3	1	0	4	Unigene_0848
		PM23	1	1	1	3	Unigene_0999
		PM36	2	1	0	3	Unigene_1204

RAB^d^	RAB21		85	52	101	238	Unigene_0008
	RAB24		64	29	68	161	Unigene_0017
	RAB16	RAB16b	46	27	66	139	Unigene_0021
		RAB16c	27	11	26	64	Unigene_0047
		RAB16d	1	4	6	11	Unigene_0247
	RAB25		45	13	52	110	Unigene_0028

^a^Cytochrome P450 family, ^b^heat shock protein, ^c^late embryogenesis abundant protein, and ^d ^RAB protein.

### DEGs between *LYP9 *and its parents

**Figure 6:**
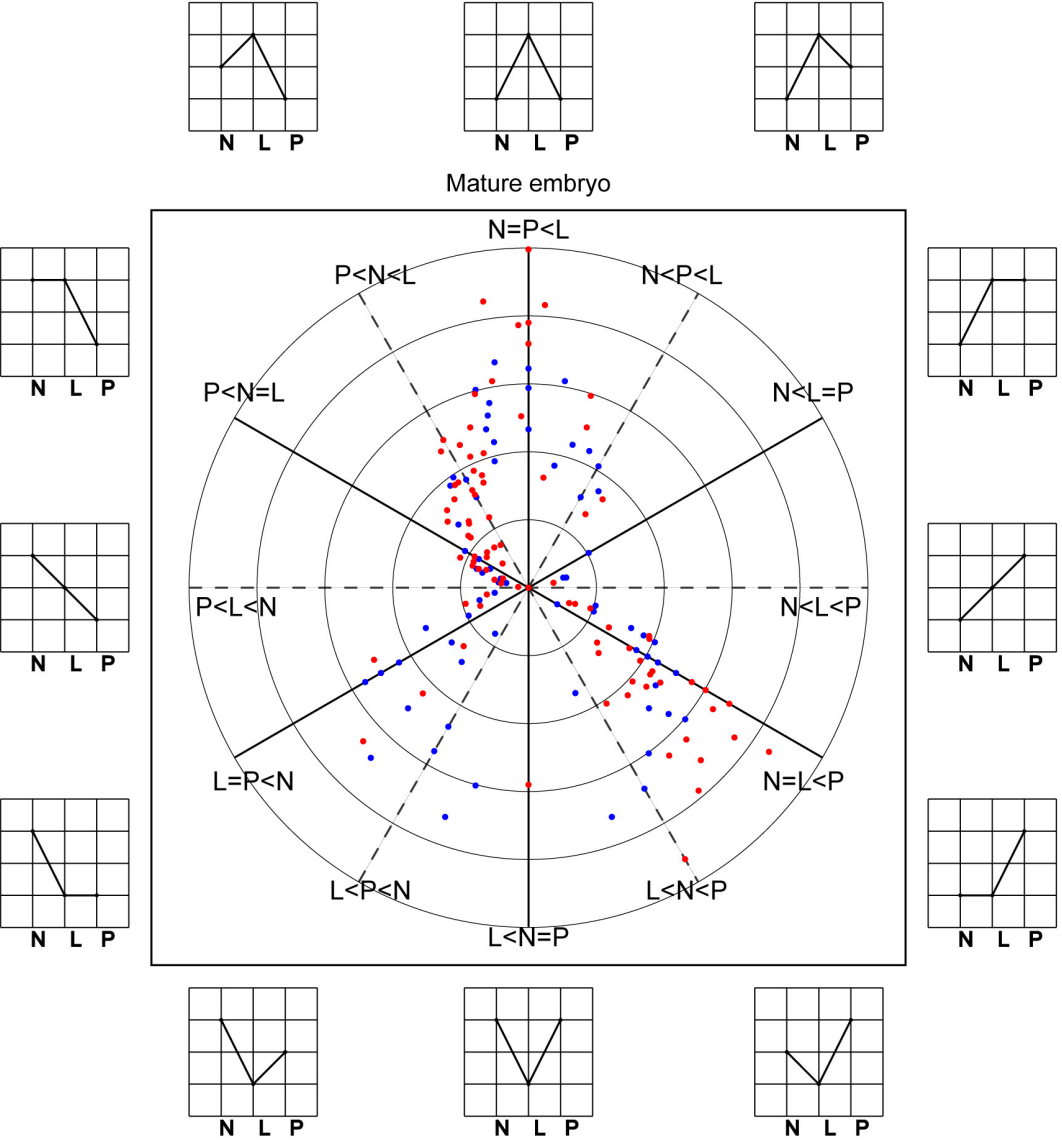
**Distribution of DEGs by fold changes**. A two-dimensional display of expression profiling and fold changes of DEGs in mature rice embryos. N, L, and P stand for *93-11*, *LYP9*, and *PA64S*, respectively. 12 different expression projections are displayed clock-wise according to their fold changes. The radius at which a gene is plotted represents fold changes L/[(P+N)/2]. Spots falling on the horizontal and vertical lines exhibit pure additivity and over- (or under-) dominance, respectively.

Based on P-values of 0.05 and 0.01 from general Chi-squared test, we defined 191 (2.53%) and 89 (1.12%) DEGs, respectively, between *LYP9 *and its parental lines (Table 7). The full list of all DEGs is in Additional file 4: DEGs and their expression patterns. Most of these DEGs belong to high- and medium-abundance genes, 84.3% and 59.2%, respectively. We focus our discussion on the larger group (P-value 0.05) unless specified. The DEGs cover almost all expression patterns with rather unequal distributions. First, the majority of DEGs (84.3%, 161) exhibit either high- or low-parent dominances rather than deviate significantly from both (15.7%, 30). Second, of the 161 genes, 72.7% (117) show differences between *PA64s *and *LYP9*, whereas only 27.3% (44) exhibit differences between *93-11 *and *LYP9*. This result indicated that the gene expression pattern of *LYP9 *is more similar to *93-11 *than to *PA64s *at this developmental stage. Third, there are slightly less high-parent dominant genes (76) than low-parent dominant ones (85) in embryo both between *93-11 *and *LYP9 *(18/26) and between *PA64s *and *LYP9 *(58/59), whereas over-dominant genes (20) are more than under-dominant genes (6). Finally, there are slightly more up-regulated genes than down-regulated genes in general. When we used the more stringent P-value (0.01), most of the observed rules did not change except that the up-regulated genes between *PA64s *and *LYP9 *were found slightly more than down-regulated genes (36/28). In general, the distribution pattern of DEGs in the embryo is similar to that of the panicle as compared to our previous SAGE study [10]. When we displayed the fold change of DEGs (*LYP9*/[(*93-11*+*PA64s*)/2]) in a two-dimensional plot (Figure 6), the consistency remains.

**Table 7:** Differentially-expressed genes between F1 hybrid (*LYP9*) and its parental lines (*93-11 *and *PA64s*)

Genetic class	Subclass	Expression pattern	P-value(total number of DEGs)			
			
			0.05 (191)		0.01 (89)	
			
			#	%	#	%
Up-regulated	Over-dominance	L > N > P	5	2.6	3	3.4
		L > P > N	0	0	0	0
		L > N = P	15	7.9	8	9
	High-parent dominance	L = P > N	18	9.4	6	6.7
		L = N > P	58	30.4	36	40.4
Additivity	Additivity	N < L < P	1	0.5	0	0
		P < L < N	3	1.6	0	0
Down-regulated	Low-parent dominance	L = P < N	26	13.6	7	7.9
		L = N < P	59	30.9	28	31.5
	Under-dominance	L < N < P	2	1	0	0
		L < P < N	0	0	0	0
		L < P = N	4	2.1	1	1.1

N, P, and L stand for paternal lines (*93-11*), maternal lines (*PA64s*), and F1 hybrids (*LYP9*), respectively.

### Integrated analysis with proteomic and SAGE data

We compared our DEGs to those identified from proteomic data generated from the same material (Weiwei Wang, personal communication). The proteomic data were acquired from the same material as what we used in this study, mature embryo of *93-11*, *PA64s*, and *LYP9*. Over 1,300 2-D gel spots (putative proteins) were analyzed and 54 differentially-expressed proteins were identified in the study. We found that most of the high-abundance genes also highly expressed in the proteomic data, but the expression patterns were not always consistent. There were only nine genes found consistent in both datasets, and three of them were not (Table 8, when there are several protein spots for one gene and one of the spots are consistent with the EST data, we classified this gene as consistent). Seven of the consistent DEGs were found expressed higher in *LYP9 *except early embryogenesis protein and glutelin; both are down-regulated. Most of the DEGs in the protein data fell into three general categories: additivity, over-dominance, and under-dominance, whereas most of the DEGs generated from the EST data are in other groups: high- and low-parent dominance. We also compared our EST data to those of our SAGE experiments generated from the same hybrid triad. The SAGE data were generated from nine SAGE libraries made from three tissues: root, leaf, and panicle of the same hybrid triad, *93-11*, *PA64s*, and *LYP9*. Roughly ten-thousand SAGE tags from each library were obtained and 20,595 unique tags were annotated, and among them, 1,216 DEGs (p < 0.01) were detected [19]. In this study, we identified 22 DEGs between the two datasets, which are shared by at least one tissue (library). The expression patterns in both dataset were summarized in Additional file 5: DEGs shared with SAGE data. These genes are more likely to be associated with heterosis, especially when some of the DEGs have consistent expression pattern in all tissues.

**Table 8:** DEGs shared by EST and protein data

Unigene ID (description)	EST(copy number)			Protein (vol%)		
		
	*93-11*	*PA64s*	*LYP9*	*93-11*	*PA64s*	*LYP9*
Consistent						

Unigene_0007 (LEA3)	90	61	98	0.962	-	0.326
Unigene_0012 (sHSP)	28	74	101	0.323	0.721	0.782
Unigene_0025 (Bowman Birk trypsin inhibitor)	49	13	49	0.413	0.571	0.856
				2.64	0.643	0.808
				0.835	0.217	0.203
Unigene_0054 (Lipoprotein)	12	4	35	0.896	1.18	2.33
				0.321	0.741	-
				0.462	1.14	0.751
Unigene_0006 (Lectin)	106	53	97	0.952	0.544	0.211
				0.121	0.158	0.405
Unigene_0299 (Enolase)	0	5	4	0.421	0.595	0.736
Unigene_0048 (Embryo-specific protein)	21	9	33	0.624	0.883	1.67
Unigene_0073 (Early embryogenesis protein)	26	3	9	0.145	0.136	0.298
				0.286	-	0.197
				0.392	0.067	0.073
Unigene_0442 (Glutelin)	1	6	0	0.154	0.319	0.824
				0.202	0.732	0.261
				0.242	0.38	-

Not Consistent						

Unigene_0093 (PreproMP73)	8	5	17	0.184	0.146	-
				0.061	0.303	0.104
Unigene_0011 (HSP70)	71	50	88	0.421	0.923	0.332
Unigene_0253 (Superoxide dismutase)	1	8	2	0.434	-	0.301

### Alignments of DEGs to genome sequences

To understand the regulatory mechanism of DEGs, we traced their genomic sequences in 93-11 and *PA64s*, and identified the regulatory and transcript sequences among orthologous gene pairs. We scrutinized the genomic sequences (3 kb upstream from the translational start codon including 5'UTR, 3 kb downstream from the translational stop codon including 3'UTR, and coding region) from the genome sequences of *93-11 *and *PA64s *for the 22 DEGs that are confirmed with our SAGE data. We found that 9 (41%) genes have almost identical sequences in all regions (without deletion/insertions more than 3 bp in length), and we believed that these genes might be regulated by distant trans-regulatory mechanisms other than cis-elements in their immediate promoters. Among the 13 (59%) genes that showed significant sequence deviations, 5 (23%), 10 (45%), and 11 (50%) genes have differences in coding, 5'UTR, and 3' UTR regions, respectively. We also analyzed well-defined regulatory sequence motifs and found that 14 (64%) genes have obvious motif variations. Our results were summarized in Table 9.

**Table 9:** DEGs in parental genomes

*93-11*	*PA64*s	5'UTR	Coding	3'UTR	Motif
9311_Chr01_0275	Pa64_Chr01_0330	N	N	N	Y
9311_Chr02_0049	Pa64_Chr02_0052	N	N	N	N
9311_Chr02_2243	Pa64_Chr02_2903	N	N	N	N
9311_Chr03_0335	Pa64_Chr03_0342	N	N	N	N
9311_Chr03_1553	Pa64_Chr02_1741	N	N	N	N
9311_Chr03_2170	Pa64_Chr02_1375	N	N	N	N
9311_Chr05_2916	Pa64_Chr05_2748	N	N	N	N
9311_Chr05_2972	Pa64_Chr05_2805	N	N	N	N
9311_Chr07_1714	Pa64_Chr07_1619	N	N	N	N
9311_Chr01_4347	Pa64_Chr01_4085	N	N	Y	Y
9311_Chr03_0234	Pa64_Chr03_0241	N	N	Y	Y
9311_Chr10_1957	Pa64_Chr10_1617	N	N	Y	Y
9311_Chr01_2124	Pa64_Chr01_2174	Y	N	N	Y
9311_Chr11_1172	Pa64_Chr11_1013	Y	N	N	Y
9311_Chr05_0084	Pa64_Chr05_0081	Y	N	Y	Y
9311_Chr07_0583	Pa64_Chr07_0573	Y	N	Y	Y
9311_Chr11_0059	Pa64_Chr11_0066	Y	N	Y	Y
9311_Chr01_0387	Pa64_Chr01_0451	Y	Y	Y	Y
9311_Chr03_0613	Pa64_Chr03_0602	Y	Y	Y	Y
9311_Chr03_1065	Pa64_Chr03_1056	Y	Y	Y	Y
9311_Chr04_0532	Pa64_Chr03_0450	Y	Y	Y	Y
9311_Chr06_3185	Pa64_Chr06_2801	Y	Y	Y	Y

N stands for insignificant differences (less than 3 bp in the sequence) and Y stands for significant differences (more than 3 bp in the sequence or motif differences).

### Quantitative PCR-based Experimental validation of DEGs

**Figure 7:**
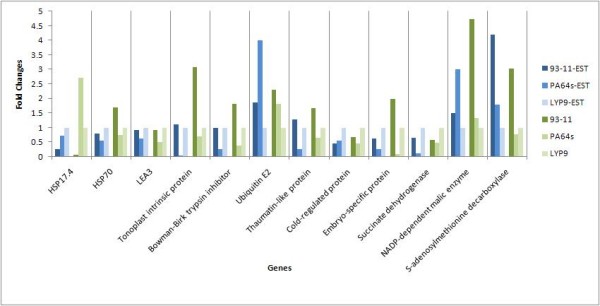
**qRT-PCR verification**. The quantitative real-time PCR results confirmed the expression variations among samples. The EST section showed the fold changes detected by cluster size.

We chose eight stress-tolerance-associated and four development-associated genes–both expressed in high-abundance and functionally important to mature rice embryo–to validate their expression patterns, using quantitative real-time PCR or qRT-PCR. The stress-tolerance-associated proteins are LEA3, HSP17.4, HSP70, tonoplast intrinsic protein, ubiquitin E2, thaumatin-like protein, Bowman-Birk trypsin inhibitor, and cold-regulated protein (gi| 115464405|). The four development-associated genes included one embryo-specific protein and three metabolism-associated proteins (succinate dehydrogenase, NADP-dependent oxidoreductase and S-adenosylmethionine decarboxylase). Almost all the results showed consistent trends with their corresponding EST data (Figure 7). We listed the primer sequences used in the experiment in Additional file 6: Primers for qRT-PCR.

## Discussion

### Genes in abundance: stress tolerance and development

High-abundance genes are known to characterize tissue- or organ-specificity since they are dominant in manifestation of major functions of the tissue or organ. We found that the embryo shares 3,649, 3,670, and 2,768 genes with leaf, panicle, and root, respectively, and there are 2,342 genes common to all tissues. All high-abundance and most medium-abundance genes in our dataset are either specifically expressed or unique to their tissues of origin whereas only 47 genes in medium-abundance expressed universally at similar expression level, as compared to the SAGE data, distributing among the most basic GO categories (Additional file 7: GO classification of universally-expressed genes among embryo and other tissues from SAGE data.). In an attempt to define functional characteristics of the mature rice embryo based on its high- and medium-abundance genes, we found that two major functional categories stood out: stress tolerance and development, which are composed of 56.3% and 9.4% of high-abundance genes, respectively. Furthermore, there are still large amount of genes in the universally expressed categories such as metabolism and cellular process which expressed specifically in high-abundance in mature embryo.

We identified a large number of genes that are in the category of anti-stress via various mechanisms, which include a complete water-deficit tolerance system and genes involved in resistance in oxidation damage and fungal pathogen infection (Table 6 and Table 10). Water deficit is a major threatening factor to embryos, and a water-deficit tolerance system in plants was reported, including membrane proteins, chaperones (mainly HSPs and LEAs), water channel proteins, protease inhibitors, proteases, and the ubiqutin system [17]. The three high-abundance gene families (LEA, HSP and RAB) that we categorized in this study all have important functions in this system [17,18]. Our proteomic study with the same material also provided a footnote to this conclusion (Weiwei Wang, personal communication).

**Table 10:** Stress-associated genes

Water-deficit Tolerance Genes						
Protection	Name (Unigene ID)		Expression level			
			
			*93-11*	*PA64s*	*LYP9*	Total
	
	WSI	WSI18 (Unigene_0040)	28	6	43	77
		WSI76 (Unigene_0061)	12	6	22	40
	Tonoplast intrinsic protein	1.1 (Unigene_0034)	50	2	45	97
		3.1 (Unigene_0074)	16	5	16	37
		1.2 (Unigene_0378)	1	1	5	7
	Aquaporin PIP2.1 (Unigene_0260)		4	1	6	11
	Water channel protein (Unigene_0100)		12	10	7	29
	Bowman Birk trypsin inhibitor (Unigene_0025)		49	13	49	111
	Lectin (Unigene_0006)		106	53	97	256
Degradation	Ubiquitin E2 (Unigene_0018)		41	88	22	151
	Cysteine proteinase (Unigene_0051)		17	16	24	57
	Aspartic proteinase oryzasin-1 (Unigene_0176)		8	5	2	15
	Aspartic protease (Unigene_0161)		5	6	5	16
	Asparaginyl endopeptidase REP-2 (Unigene_0083)		8	20	5	33

Other Abundantly Expressed Stress-associated Genes						

Protection	Name(Unigene ID)		Expression level			
			
			*93-11*	*PA64s*	*LYP9*	Total
	
	Thaumatin-like protein (Unigene_0009)		111	22	86	219
	Gytochrome P450 monooxygenase (Unigene_0001)		1111	348	1280	2739
	NAD-/NADP-dependent oxidoreductase (Unigene_0013)		61	25	111	197
	Heavy metal transport/detoxification protein (Unigene_0107)		4	9	14	27
	Universal stress protein (Unigene_0238 and Unigene_0234)		4	4	4	12
			7	2	3	12

Three gene families–Em, Ec, and germin proteins–have been suggested to regulate the development of rice embryo to seedling [15]. The Em and Ec proteins are encoded by conserved mRNAs stored in mature embryos but germin transcribes after growth recovery. The Em protein is involved in the process of seed desiccation, and the Ec protein is the only *bona fide *Zn metallothionein yet found in higher plants [15]; both may play important roles in ion homeostasis during embryogenesis since Em and Ec proteins are expressed as the most abundantly expressed genes in our dataset with 379 and 138 ESTs, respectively. In addition, we identified one embryogenic cell protein 40 in the high-abundance gene class as well as other development-associated proteins including embryo-specific protein, differentiation embryo protein 31, and early embryogenesis protein in medium-abundance genes.

Pathway analysis on the mRNA repertoire provided a better glance on the basic physiology of rice mature embryos detected by pathway analysis, which showed that the embryo is well-prepared for rapid development. First, genes in the pathways of metabolism, especially the subsets of carbon, energy, and amino acid metabolisms, are stored in high abundance. Material utilization and energy production, such as glycolysis (10.4% of carbon metabolism), TCA cycle, starch and sucrose metabolism, oxidative phosphorylation, and carbon fixation are of essence for the embryo. Second, genes participating in translation (43.4% of GIP) and sorting (39.0% of GIP) are in high abundance to ensure rapid translation of the reserved RNA. Third, in the category of EIP, signal transduction (58.1% of EIP) and membrane transport (41.9% of EIP) stood out obviously to guarantee rapid signal transduction in the recovery phase, especially the MAPK signal pathway that is important in regulating cell cycle. Fourth, the defence (endocrine and immune) system is the highly-expressed gene category in our dataset for cellular processes, which ensures hormonal regulation in development and protection.

Our detailed analyses indicated that several important pathways active in mature plant tissues are almost absent in embryos. For example, we only identified nine ESTs involved in photosynthesis and none involved in respiration from our dataset. Although present at low level, transcription (11.1% of GIP) and replication/repair (6.4% of GIP) genes are important for growth recovery as compared to the complete absence of genes in the categories of signal molecules and interaction as well as the low level of cellular process genes in our dataset. The reservoir of the reserved mRNAs in the mature embryo appeared well-prepared for basic metabolism, response to environmental stimulus, and signal transduction to ensure rapid recovery. In the contrary, secondary metabolism, cellular process, and transcription constitute a relatively small portion of the total, and most of these functions must depend upon new mRNA syntheses.

### Possible biological mechanisms of heterosis

**Figure 8:**
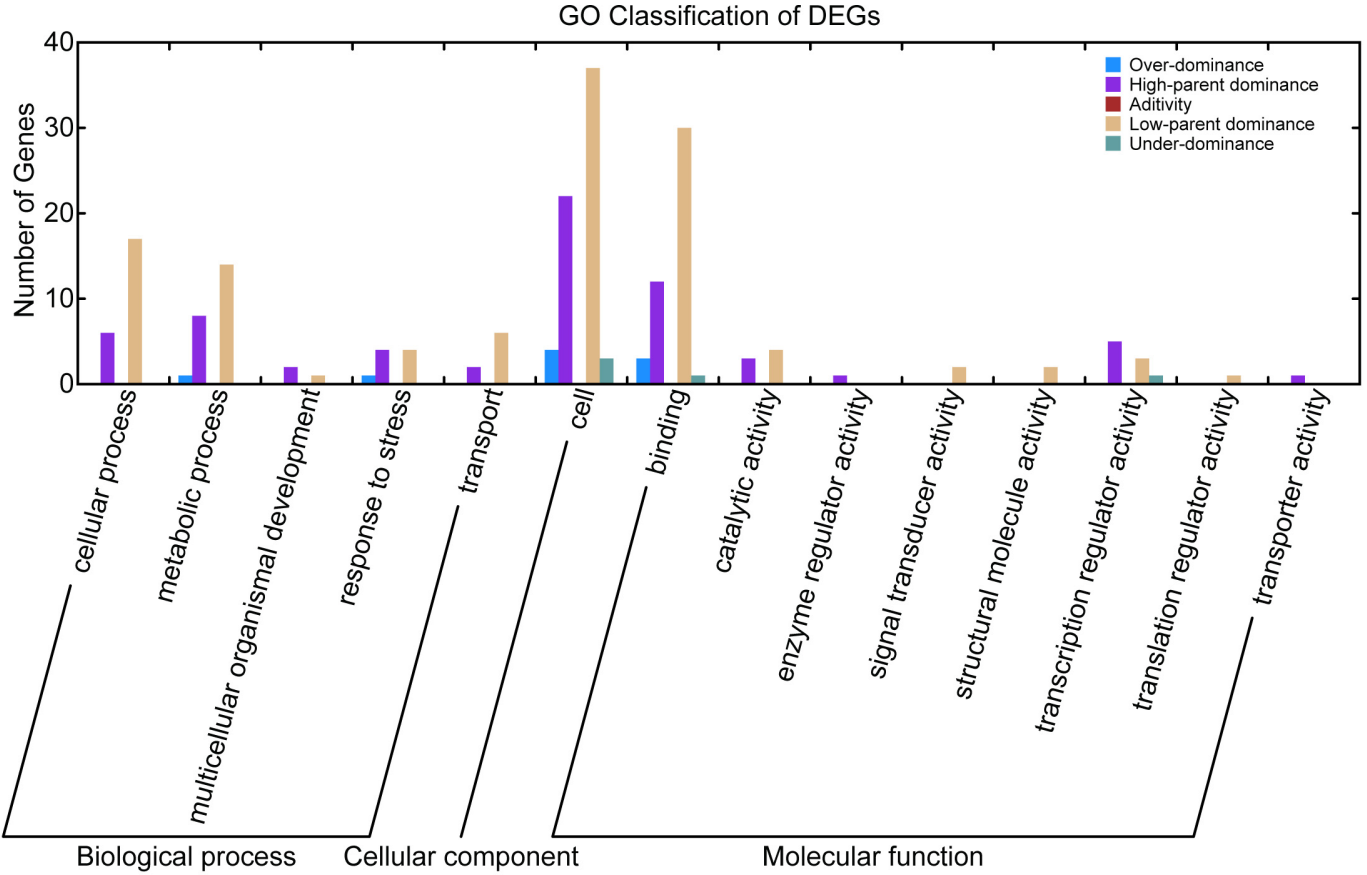
GO classification of DEGs.

When displaying all DEGs into two-dimension space, we found a composite expression pattern that suggests multiple modes of action as they are classified into 5 patterns: over-dominance, high-parent dominance, additivity, low-parent dominance and under-dominance (Table 7). Although up- and down-regulated DEGs are almost equal in numbers (Figure 6), up-regulated genes are dominant in the most abundant gene classes including cellular, metabolic, response to stress, embryo development, enzyme regulator activity, transcription regulator activity, and transporter activity (figure 8).

### Strengthened stress-tolerance system in F1

**Figure 9:**
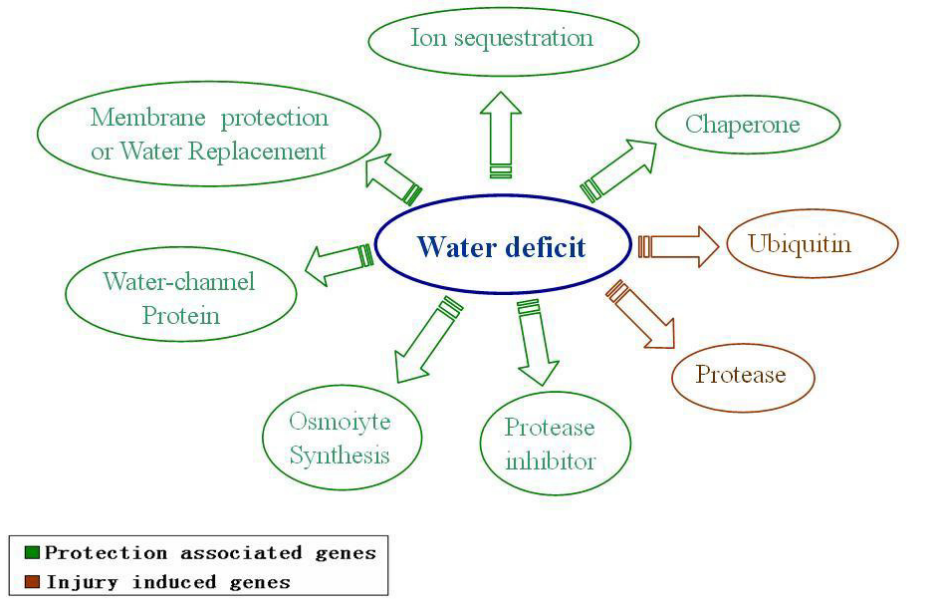
**DEGs in water-deficit tolerance system**. Note that genes playing protective roles are all up-regulated whereas those injury-induced are down-regulated.

The majority of stress-tolerance genes in our dataset exhibited differential expressions among the hybrid triad, and especially showed up-regulated expressions in *LYP9 *(Table 6 and Table 10). In the GO term, five out of seven (71.4%) DEGs are in the category of response to stress and up-regulated in F1. In the gene families related to stress tolerance (HSP, LEA, and RAB), 70.4% were differentially expressed whereas 89% of them are up-regulated together with 5.5% down-regulated and 5.5% additivity in F1. And as shown in Table 10, among 65% of the DEGs, 77% of them are up-regulated (protective genes) as opposed to 23% down-regulated (the degradation genes). Functionally, stress-tolerance genes are classified into two groups: protective and injury-induced genes. Taking the water-deficit-tolerance related genes as examples, we know that protective genes, including chaperones (mainly LEAs and HSPs), protease inhibitors, water-deficit induced membrane proteins, and water channel proteins, are counterparts of the protein-degrading mechanism, promoting survivability under water-deficit conditions. Injury-induced genes include proteases and ubiquitin system, which are involved in the degradation of proteins that are denatured during cellular water loss (Figure 9). In *LYP9*, most of the protective genes are up-regulated as compared to the down-regulated injury-induced genes. Protective genes that participate in other stress-related functions are also up-regulated in the F1. Proteomic data also added footnotes to this notion, where the most functionally important and representative genes, including LEA3, sHSP, Bowman-Birk trypsin inhibitor, Lipoprotein and lectin, thaumatin-like protein were found as up-regulated DEGs. In addition, our SAGE data also pointed out a universal strengthening in the ability of stress-tolerance in the F1, where heavy metal transport/detoxification protein, senescence-associated protein and RAB21 protein were found up-regulated albeit in other tissues of *LYP9*. We validated some of these results with qRT-PCR.

### Better preparation for growth recovery

DEG analysis suggested that the F1 appears better prepared for growth recovery as compared to its parental lines. First, genes participating in material and energy utilization are up-regulated and genes participating in anabolism are down-regulated (Additional file 8: Summary of DEGs in pathway analysis). In general, the up-regulated DEGs were found in carbon metabolism with an exception of the oxidative phosphorylation of a NADP-dependent malic enzyme in energy metabolism. In contrast, genes in carbon, nitrogen fixation, and nucleotide metabolism are mostly down-regulated. In amino acid metabolism, we identified genes both up-regulated and down-regulated, but all genes related to amino acid biosynthesis are down-regulated. This indicated that *LYP9 *up-regulate material and energy generation and utilization to ensure its embryo to be active immediately when being re-hydrated as material anabolism is normally down-regulated. Second, in the category of GIP, genes involved in protein folding and associated processing are up-regulated and genes of ubiquitin mediated proteolysis and proteasome are down-regulated. Genes involved in transcription are down-regulated but those involved in translation are not obviously biased, where both down- and up-regulated genes were found. Third, in EIP, genes in membrane transport and MAPK signal transduction are up-regulated but calcium signalling pathway and phosphatidylinositol signalling systems are down-regulated; the result suggested that MAPK signalling pathway may be the major signal pathway triggered as development starts after re-hydration. Finally, DEGs involved in cellular process but not in the response (immune) system appeared down-regulated. Some of these genes and their trends of expressions were found consistent in our SAGE and proteomic data. For example, the up-regulation of an important enolase was confirmed in the protein data. And the up-regulation of succinate dehydrogenase was validated based on the qRT-PCR test. Another instance is β-amylase that is a key enzyme for starch hydrolysis, which is not only found up-regulated in the embryo but also in the leaf and panicle of *LYP9*.

### Complementation and over-dominance

We found that embryo-characteristic genes among DEGs are primarily up-regulated and showed high-parent dominance (79.2%) with the addition of over-dominance (20.8%). The high-parent dominance genes often occur in gene families. For example, in the HSP family, the expression of LMW HSPs has the tendency of *PA64s*, higher than that of *93-11*, or expressed in an over-dominance fashion. In contrast, the expression level of classical HSPs resembles that of *93-11*, which is higher than that of *PA64s*, exhibiting over-dominance. In the LEA and RAB family, as well as other stress-tolerance genes, all the DEGs show high-parent dominance with expression levels similar to *93-11*. More than two third of the DEGs differ between *PA64s *and *LYP9 *(72.7%), whereas less than one third show differences between *93-11 *and *LYP9 *(27.3%). The result is consistent with the fact that *LYP9 *is more like *93-11 *than *PA64s *in their phenotypic appearances (Lihuang Zhu and Qingzhong Xue, personal communications). Therefore, it appears that the hybrid vigour of *LYP9 *may be contributed by a composite of beneficial alleles from both parents, complementing the relatively weaker alleles in a global fashion, and over-dominance is an essential strategy for stress tolerance and metabolism.

### Strengthening tissue-characteristic functions may contribute to heterosis

Analyses on DEGs have indicated that the mode of action for heterosis is most likely multi-fold and at multiple levels. If we have to point out a single mode of action, we believe that the strengthening of embryo-characteristic genes in *LYP9 *is most noticeable. For instance, the F1, *LYP9*, is predicted to have a significantly better stress-tolerance system than its parental cultivars, supported by up-regulated protective genes and down-regulated injury-induced genes. In addition, *LYP9 *keeps strong responses to environmental signals and potentially high-level activities after growth recovery. Therefore, when seeds re-hydrate, the F1 embryo may perform better than its parental lines. This phenomenon can be cautiously generalized based on similar analyses among other tissues of the same hybrid line. For example, in leaf, it was detected that up-regulated genes in hybrid are dominated by DEGs related to enhancing carbon assimilation whereas those related to decomposition of carbohydrates are down-regulated [10], resulting in an increased efficiency of photosynthesis or energy production.

## Conclusion

We reported a comparative transcriptomic analysis based on ESTs acquired from mature embryos of a rice hybrid and its parental lines. Our study not only provided an initial characteristic description of expression profiles of rice embryos but also pointed out the enrichment of genes involved in stress-tolerance and preparation-for-recover (both development and physical activity). We also identified 191 DEGs between F1 and its parental lines. Functional analyses revealed clues for rice heterosis, such as multiple modes of actions and strengthening of embryo-characteristic function. Genes involved in stress-tolerance and preparation-for-recovery may play key roles for rice heterosis in mature embryos.

## Methods

### Plant materials and cDNA libraries

A fresh-harvest of mature seeds for *LYP9 *(F1 embryo harvested from the maternal plant,*PA64s*) and its parental lines *93-11 *and *PA64s *were collected and stored at room temperature. The seeds were provided by the Chinese National R&D Center on Hybrid Rice, Changsha, China, and the plants were grown in its experimental rice paddies (28°30N, 113°40E, and altitude 40 m). Mature rice embryos were separated manually from the seeds by using scalpels and stored in liquid nitrogen immediately until RNA isolation.

We constructed cDNA libraries for each rice cultivar, using a combined priming method, i.e. used both oligo-dT- and random-priming to avoid potential cloning biases. Briefly, total RNA was extracted from dissected embryos with Trizol (Invitrogen), and mRNAs was purified with Oligotex mRNA Midi Kit (QIAGEN). Reverse transcription was carried out separately by using oligo-dT (with XhoI-linker sequence) and random primers in equal amount. For oligo-dT-primed cDNA, the second strand was synthesized and linked to an EcoRI-linker. After XhoI digestion, insert DNA was fractioned (1–2 Kb) with QIAquick Gel Extraction Kit (QIAGEN) and cloned into a directional pBluescript^® ^II XR vector (Stratagene). For random-primed cDNA library, fractioned cDNA was cloned into a pUC18 vector.

### EST assembly and annotation

We picked approximately 7,000 clones from each oligo-primed library and over 3,000 clones from each random-primed library for single-pass unidirectional sequencing from the 5'-end (MegaBase^® ^1000 sequencers). We used Phred [20,21] for base-calling, rejected low-quality sequences according to default settings, and removed low-quality sequences shorter than 100 bp. Cross_match program was used to trim vector sequences. We also removed ESTs that do not have significant similarities [22] with the collection of Oryza sativa Gene Index database (release_17, it contains the largest assembly of publicly available rice ESTs) and the reference genomes including predicted genes from *93-11 *[11]. We used Phrap (minmatch 50 and minscore 100) and Consed [23] to build consensus sequences and to view the assemblies. We further aligned all unigenes to NCBI nr-databases and clustered unigenes with the same gi-numbers to form non-redundant unigenes after manual inspections. In addition, we calculated the expression abundance for all unique sequences or unigenes based on ESTs.

We first annotated our data based on sequence similarity searches, using blast-based tools [22] (tblastx) against several databases, including NCBI non-redundant protein database, two public rice genome annotation databases (BGI rice genome annotations and TIGR rice pseudomolecule release 5), two major rice EST assembly databases (NCBI UNIGENE release 62 and TIGR OGI release 17) with an e-value cutoff of 1e-5. We then assigned functional categories for the unigenes according to GO functional classification using a web-based tool WEGO [24] and biological processes/pathways using the online KEGG Automatic Annotation Service  with the BBH option checked.

### Defining specifically-expressed and differentially-expressed genes

We selected eight representative libraries from the NCBI Digital-Differential-Display (DDD) database for a comparative analysis (Table 5). We measured gene expression abundance as transcript per million (TPM) and sorted the expression abundance and annotation for every unigene among different tissues. Then do the hierarchical clustering analyses using default parameters. The parallel analysis with SAGE data was based on rice genome annotations with similar parameters. The GO classification analysis on both predicted genes from the parental (*93-11 *and *PA64s*) genomes and the mature embryo were performed and categories that has a larger percent of genes in embryo than genome were supposed to be enriched than genome general transcriptome.

DEGs among the three cultivars were determined by using IDEG6 [25,26]. The cutoff value was set to p <= 0.05 for general Chi-squared test. All DEGs were classified into twelve offspring-parent distribution modes according to their expression patterns. Fold changes were detected based on the equation *LYP9*/[(*93-11*+*PA64s*)/2]. The radius at which a gene is plotted represents fold change and the position where each spot laid depends on the expression relationship among three libraries.

### Quantitative Real-time PCR validation

We selected 12 functionally important and representative DEGs for validation using quantitative real-time PCR (qRT-PCR). Total RNA was digested with RNase-free DNase I to remove DNA contaminations and reverse-transcribed with a mixed primers (oligo-dT primer: random primer in a molar ratio of = 7:3). Gene-specific primers were designed for each DEG. and the rice β-actin gene was used as a control. qRT-PCR was performed by using a Quant SYBR Green PCR kit (Tiangen, china). The results were based on the average of three parallel experiments and analysed with the Opticon Monitor software. Melting curves and CT (cycle threshold) values for DEGs were used to measure expression levels. We calculated standard deviations for parallel samples, set cut-off standard to one, and quantified gene expression based on normalization with the control and self-normalization in parallel samples.

## Authors' contributions

XG constructed the cDNA libraries, prepared clones for sequencing, performed data processing, functional analyses of ESTs, and qRT-PCR as well as drafted the manuscript. WC provided guidance in data processing and analysis. WW and SS helped in organizing and revising the manuscript. JY and SH supervised the study and revised the manuscript. All authors read and approved the final manuscript.

## Supplementary Material

Additional file 1 **Summary of unigenes.** We summarized expression abundance and distribution in three libraries of all unigenes as well as their best hits in NCBI nr database (NA: no hit). Click here for file

Additional file 2 **A full list of unigene sequences.** We listed all unigene sequences assembled from ESTs. Click here for file

Additional file 3 **Hierarchical clustering of medium-abundance genes.** We clustered medium-abundance genes to show that its majority are also enriched in mature rice embryo as compared to other representative tissues chosen from NCBI DDD database (A) and the SAGE data from the same hybrid line (B and C). Click here for file

Additional file 4 **DEGs and their expression patterns.** We provided the unigene ID, expression pattern, and P-value for each DEG. N, P and L stand for 93-11, PA64s and LYP9 respectively. A hyphen denotes the absence of expression in EST data. Click here for file

Additional file 5 **DEGs shared with SAGE data.** We marked the expression pattern of DEGs in other tissues with up and down arrows. A hyphen denotes the absence of differential expression. Click here for file

Additional file 6 **Primers for qRT-PCR.** We provided primer sequences used in qRT-PCR. Click here for file

Additional file 7 GO classification of universally-expressed genes among embryo and other tissues from SAGE data. We classified genes that universally expressed at similar expression level among embryo and other tissues from SAGE data based on basic categories of Gene Ontology. Click here for file

Additional file 8 **Summary of DEGs in pathway analysis.** We showed unigene ID, expression pattern, and associated pathway for all DEGs. Click here for file
