# Supplementary figures and images for: Transcriptomic profiling of mature embryo from an elite super-hybrid rice LYP9 and its parental lines
Source: BMC Plant Biol. 2008 Nov 11;8:114. doi: 10.1186/1471-2229-8-114 (PMC2596138; doi:10.1186/1471-2229-8-114)

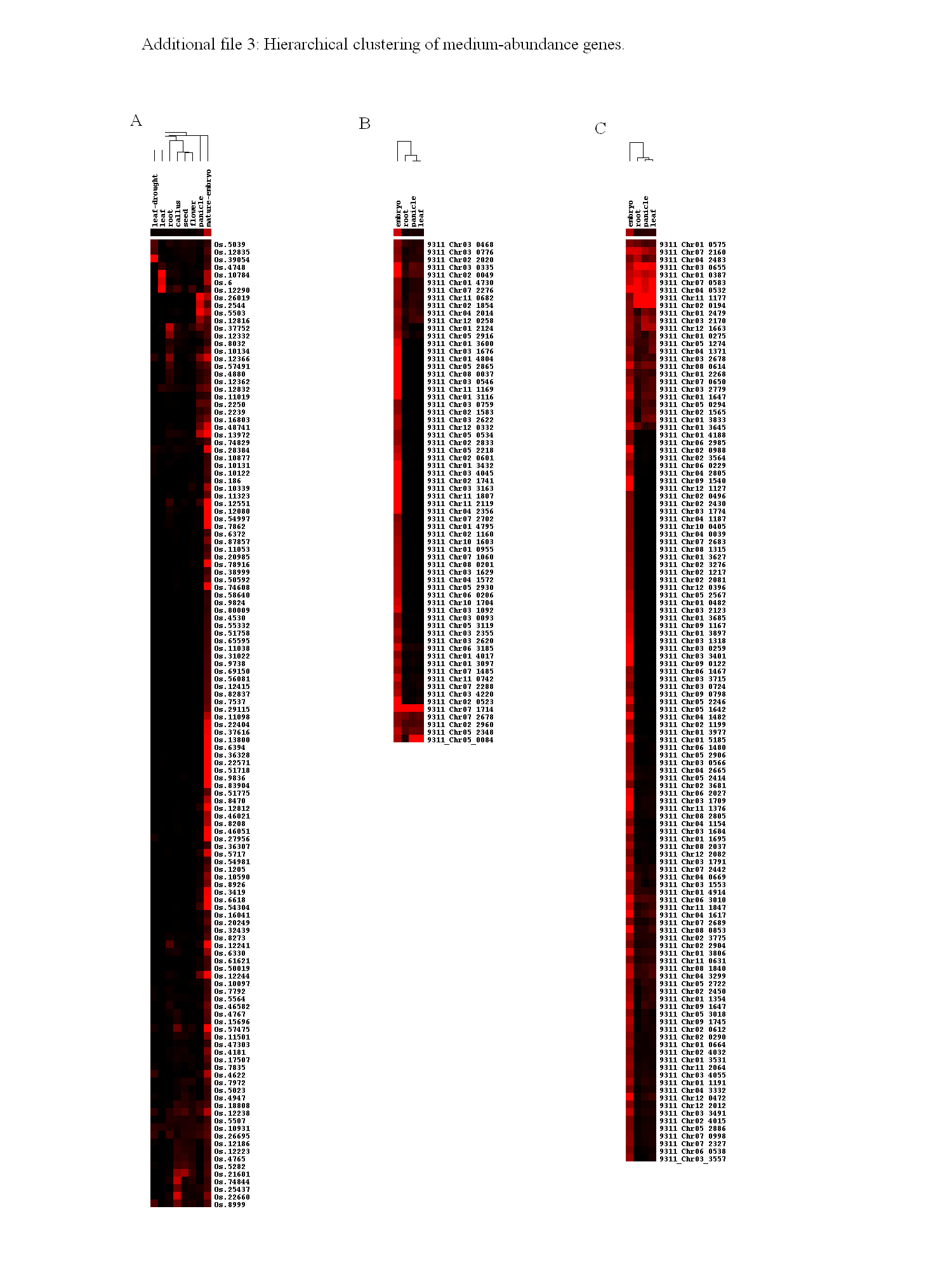

Supplement: Additional file 3 — Hierarchical clustering of medium-abundance genes. We clustered medium-abundance genes to show that its majority are also enriched in mature rice embryo as compared to other representative tissues chosen from NCBI DDD database (A) and the SAGE data from the same hybrid line (B and C). [file 1471-2229-8-114-S3.tiff]
