# Supplementary material for: Transcriptomic profiling of mature embryo from an elite super-hybrid rice LYP9 and its parental lines
Source: BMC Plant Biol. 2008 Nov 11;8:114. doi: 10.1186/1471-2229-8-114 (PMC2596138; doi:10.1186/1471-2229-8-114)

Additional file 7: GO classification of universally-expressed genes among embryo and other tissues from SAGE data

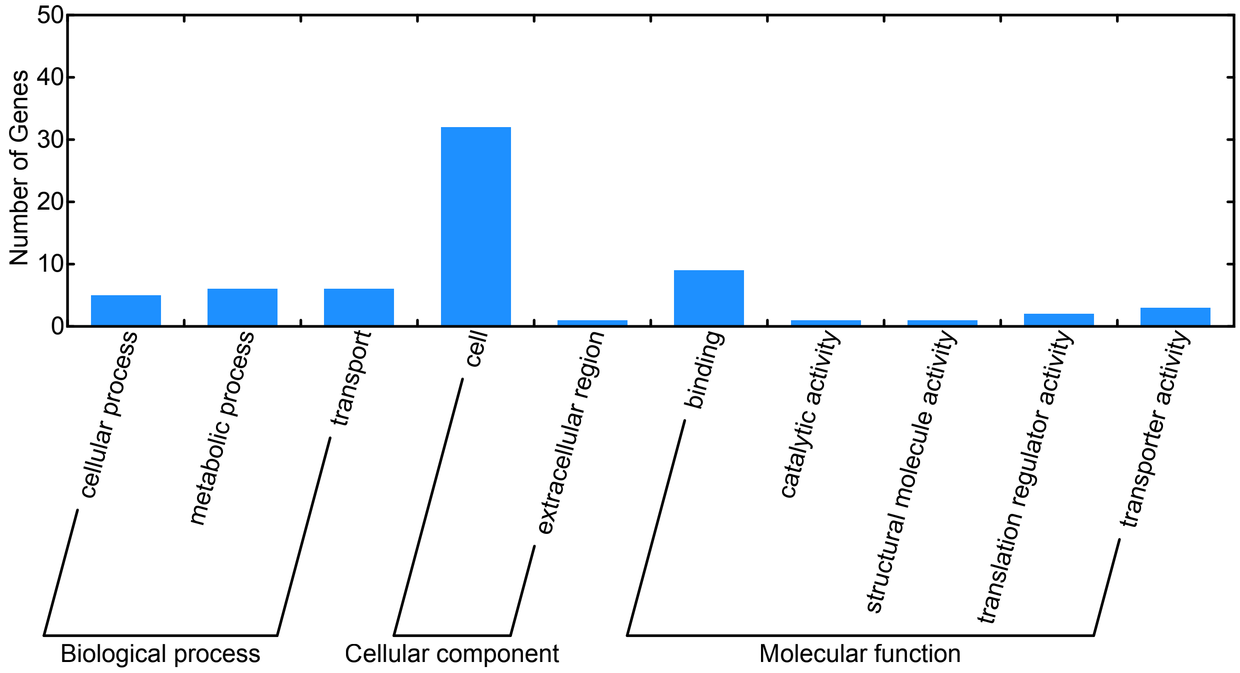

Supplement: Additional file 7 — GO classification of universally-expressed genes among embryo and other tissues from SAGE data. We classified genes that universally expressed at similar expression level among embryo and other tissues from SAGE data based on basic categories of Gene Ontology. [file 1471-2229-8-114-S7.pdf]
